# Modeling *in-vivo* protein-DNA binding by combining multiple-instance learning with a hybrid deep neural network

**DOI:** 10.1038/s41598-019-44966-x

**Published:** 2019-06-11

**Authors:** Qinhu Zhang, Zhen Shen, De-Shuang Huang

**Affiliations:** Institute of Machine Learning and Systems Biology, School of Electronics and Information Engineering, Tongji University, Shanghai, 201804 P.R. China

**Keywords:** Computational models, Computational models, Machine learning, Machine learning

## Abstract

Modeling *in-vivo* protein-DNA binding is not only fundamental for further understanding of the regulatory mechanisms, but also a challenging task in computational biology. Deep-learning based methods have succeed in modeling *in-vivo* protein-DNA binding, but they often (1) follow the fully supervised learning framework and overlook the weakly supervised information of genomic sequences that a bound DNA sequence may has multiple TFBS(s), and, (2) use one-hot encoding to encode DNA sequences and ignore the dependencies among nucleotides. In this paper, we propose a weakly supervised framework, which combines multiple-instance learning with a hybrid deep neural network and uses *k*-mer encoding to transform DNA sequences, for modeling *in-vivo* protein-DNA binding. Firstly, this framework segments sequences into multiple overlapping instances using a sliding window, and then encodes all instances into image-like inputs of high-order dependencies using *k*-mer encoding. Secondly, it separately computes a score for all instances in the same bag using a hybrid deep neural network that integrates convolutional and recurrent neural networks. Finally, it integrates the predicted values of all instances as the final prediction of this bag using the *Noisy-and* method. The experimental results on *in-vivo* datasets demonstrate the superior performance of the proposed framework. In addition, we also explore the performance of the proposed framework when using *k*-mer encoding, and demonstrate the performance of the *Noisy-and* method by comparing it with other fusion methods, and find that adding recurrent layers can improve the performance of the proposed framework.

## Introduction

Transcription factors can modulate gene expression by binding to specific DNA regions, which are known as transcription factor binding site (TFBS). Modeling *in-vivo* TF-DNA binding, also called motif discovery, is a fundamental yet challenging step towards deciphering transcriptional regulatory networks^1,2^.

In the past decades, the introduction of high-throughput sequencing technologies, especially ChIP-seq.^3^, dramatically increases the amount and spatial resolution of available data, which is helpful for the in-depth study of *in-vivo* protein-DNA binding. However, DNA sequences directly extracted from ChIP-seq cannot precisely represent TFBS since the outputs of such experiments contain a lot of noisy^4^. Thus lots of methods have been developed for precisely predicting protein-DNA binding sites, including conventional algorithms^5–9^ and deep-learning based methods^10–12^. Not surprisingly, deep-learning based methods are better than conventional algorithms at modeling protein-DNA binding. DeepBind^10^ and DeepSea^11^ were two famous deep-learning based methods, which used convolutional neural network (CNN) to model the binding preference of DNA-proteins with a superior performance over conventional methods. DanQ^12^ designed a hybrid deep neural network to quantify the function of DNA sequences, which first used a convolutional layer to detect regulatory motif features from DNA sequences, and subsequently employed a bi-directional recurrent layer to capture long-term dependencies between motif features. Soon after, a number of deep-learning based methods are proposed for modeling *in-vivo* protein-DNA binding^13–17^.

Although deep-learning based methods have achieved remarkable performance on modeling *in-vivo* protein-DNA binding, they usually overlook the weakly supervised information of genomic sequences that a bound DNA sequence may have multiple TFBS(s). In consideration of this information, Gao *et al*.^18^ developed a multiple-instance learning (MIL) based algorithm, which combines MIL with TeamD^19^, for modeling protein-DNA binding, and recently Zhang *et al*.^20^ also developed a weakly supervised convolutional neural network (WSCNN), which combines MIL with CNN, for modeling protein-DNA binding. Moreover, they are inclined to use one-hot encoding to encode DNA sequences, which means that it only considers the independent relationship among nucleotides. However, recent studies have shown that taking into consideration the high-order dependencies among nucleotides can improve the performance of modeling protein-DNA binding^21–23^. In consideration of this information, Zhou *et al*.^24^ evaluated DNA-binding specificities based on mononucleotide (1-mer), dinucleotide (2-mer), and trinucleotide (3-mer) identity, and stated that 2-mer and 3-mer may contain implicit DNA shape information and partially capture the effect of the DNA shape variation on binding. Zhang *et al*.^25^ proposed a high-order convolutional neural network, which first used *k*-mer encoding to transform DNA sequences into image-like inputs of high-order dependencies, and then applied CNN to extract motif features from these inputs.

Inspired by the above observation, we extend our previous work WSCNN from three aspects in this paper. (1) WSCNN mainly employed CNN to learn motif features from DNA sequences, and did not take into consideration the long-term dependencies between motif features. In the weakly supervised framework, therefore we add a bi-directional recurrent layer after the convolutional layer to capture the forward and backward long-term dependencies between motif features. (2) WSCNN attempted to use four fusion methods to fuse the predicted values of all instances in a bag, and then selected the best one of them as the final prediction. However, it is inconvenient for user to decide which one is better, so they have to try the four fusion methods one by one. Therefore we offer a better and more robust fusion method *Noisy-and*^26^ to replace them. (3) WSCNN, like other deep-learning based methods, used one-hot encoding to transform DNA sequences into image-like inputs. However, the relationship between nucleotides is not independent in practice. Therefore we use *k*-mer encoding to transform DNA sequences into image-like inputs of high-order dependencies, and explore the performance of the proposed framework when using dinucleotide (2-mer) and trinucleotide (3-mer) as inputs in the weakly supervised framework. In summary, the proposed framework firstly use the concepts of MIL to segment DNA sequences into multiple instances, and adopt *k*-mer encoding to transform sequences into image-like inputs of high-order dependencies, and then design a hybrid neural network to compute a score for all instances, and finally employ the *Noisy-and* method to fuse the predicted values of all instances as the final prediction of a bag. We conducted a lot of comparative experiments on *in-vivo* datasets to show that our proposed framework outperforms other competing methods. Besides, we also show the performance gain of the proposed framework when using *k*-mer encoding, and compare the performance of the *Noisy-and* method with other fusion methods, and demonstrate the effectivness of adding recurrent layers.

The rest of the paper is organized as follows. We give a detailed description of the proposed framework, and introduce the fusion method *Noisy-and* in Section II. We give a detailed analysis of the experimental results, and discuss the hyper-parameter settings in Section III.

## Methods

In this section, we give a detailed description of the proposed framework for modeling *in-vivo* protein-DNA binding. Actually, the task can be thought of as a binary classification problem that separates positive sequences (bound) from negative sequences (non-bound). The output of the network is a probability (a scalar in [0, 1]) distribution over two labels (1/0), since a binary classification problem can be addressed also through a binary output (1/0). This framework includes three stages in general: data processing, model designing, and results merging.

### Data processing

#### Segmentation

Considering the weakly supervised information of DNA sequences, thus it is reasonable to use the concepts of MIL to deal with DNA sequences. Therefore we divided them into multiple overlapping instances following the works^18,20^, which ensures that (1) the weakly supervised information can be retained, and that (2) a large amount of instances containing TFBS are generated. This method is defined as a sliding window of length *c*, which divides DNA sequences of length *l* into multiple overlapping instances by a stride *s*. A bag is composed of all possible instances in the same sequence, and the number of instances in this bag is $\lceil (l-c)/s\rceil +1$, where *s* and *c* are two hyper-parameters that need to be tuned by cross-validation. If (*l* − *c*) is not a multiple of *s*, we pad ‘0’ at the end of DNA sequences.

#### *K*-mer encoding

After segmenting DNA sequences, all instances should be transformed into image-like inputs that can be handled by CNN. One-hot encoding is a commonly-used method in deep-learning based methods, but it ignores high-order dependencies among nucleotides. In order to capture the dependencies, therefore we use the *k*-mer encoding method^25^ to transform all instances into image-like matrices of high-order dependencies. This method can be implemented according to (1):

1 $${X}_{i,j}=\{\begin{array}{rcl}1\,{\rm{if}}\,{{x}}_{i}\,\mathrm{...}\,{x}_{i+k-1} & = & {j}^{th}{\rm{base}}\,{\rm{in}}\{{4}^{k}\,k \mbox{-} {\rm{mer}}\}\\ 0\,{\rm{otherwise}} &  & \end{array}$$

where $i\in [1,\,c-k+1]$, and *c* denotes the length of instances, and *x*_*i*_ denotes a possible character from {A, C, G, T}, and *X*_*i*,*j*_ denotes a matrix generated by using *k*-mer encoding. According to the equation, we can find that one-hot encoding is a special case of *k*-mer encoding when *k* is set to 1. For example, 1-mer encoding: each nucleotide is mapped into a vector of size 4 (A → [1, 0, 0, 0]^T^, C → [0, 1, 0, 0]^T^, G → [0, 0, 1, 0]^T^, and T → [0, 0, 0, 1]^T^); 2-mer encoding: taking into consideration the dependencies between two adjacent nucleotides, and each dinucleotide is mapped into a vector of size 16 (AA → [1, 0, 0, 0, 0, 0, 0, 0, 0, 0, 0, 0, 0, 0, 0, 0]^T^, ..., TT → [0, 0, 0, 0, 0, 0, 0, 0, 0, 0, 0, 0, 0, 0, 0, 1]^T^); 3-mer encoding: taking into account the dependencies among three adjacent nucleotides, and each trinucleotide is mapped into a vector of size 64 (AAA → [1, 0, 0, 0, ..., 0, 0, 0, 0, 0], ...,TTT → [0, 0, 0, 0, ..., 0, 0, 0, 0, 0, 1]).

**Figure 1:**
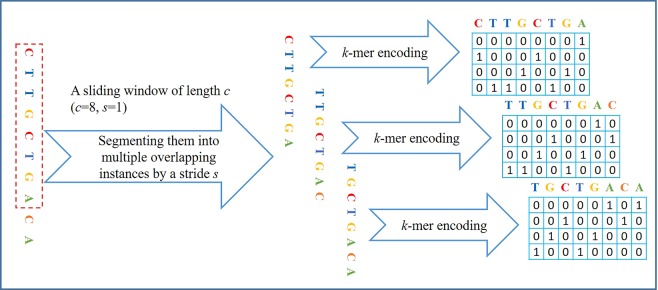
A graphical illustration of data processing when *k* = 1.

A graphical illustration of data processing when *k* = 1 is shown in Fig. 1, where *l* = 10, *c* = 8, *s* = 1, and the red dashed box denotes a sliding window of length *c* = 8. Through this stage, DNA sequences can be encoded into image-like inputs that can be easily handled by CNN.

In the implementation code, each instance is firstly encoded into a tensor of shape 1 × 4^*k*^ × 1 × (*c* − *k* + 1) (batchsize × channel × height × width), and then all instances of a bag can be concatenated along the height axis. Therefore a bag can be represented by a tensor of shape 1 × 4^*k*^ × *n* × (*c* − *k* + 1), where *n* is the number of instances per bag ($n=\lceil (l-c)/s\rceil +1$). Details of implementation can refer to our open source code.

### Model designing

Considering the spatial and sequential characteristics of DNA sequences, we design a hybrid deep neural network, which integrates convolutional and recurrent neural networks in this stage. Convolutional neural network (CNN) is a special version of article neural network (ANN)^27–29^, which adopts a weight-sharing strategy to capture local patterns in data such as DNA sequences. Recurrent neural network (RNN) is another variant of ANN where connections between neurons form a directed graph. Unlike CNN, RNN can use its internal state (memory) to exhibit dynamic temporal or spatial behavior. In the designed model, the convolution layer is used to capture motif features, while the recurrent layer is used to capture long-term dependencies between the motif features. The model is arranged in this order: a convolutional layer → a max-pooling layer → a dropout layer → a bi-directional recurrent layer → a dropout layer → a softmax layer.

#### Convolutional layer

This layer is used to capture motif features, which can be thought of as a motif scanner to compute a score for all potential motifs, and often followed by a rectified linear unit^30^ (ReLU) layer. The early work^13^ has explored the performance of using different number of convolutional kernels, and found that adding more kernels can significantly improve performance. Thus the number of kernels was set to a fixed value 16 in the proposed framework.

#### Max-pooling layer

Both DeepBind and DeepSea used a global max-pooling layer to pick out the maximum response of the whole sequence, while our deep model uses a max-pooling layer of a certain size (1, 8) to keep the local best values of the whole sequence.

#### Dropout layer

Dropout strategy^31^ is a widely-used regularization technique for reducing overfitting in deep neural networks by preventing complex co-adaptations on data, which randomly sets the outputs of the previous layer to zero with a dropout ratio. The dropout ratio is a hyper-parameter that was investigated by cross-validation in the experiments.

#### Recurrent layer

In order to capture the forward and backward long-term dependencies between the motif features, a bi-directional recurrent layer composed of long short-term memory (LSTM) units^32^ is used. A LSTM unit usually consists of a cell, an input gate, a forget gate and an output gate, where the cell remembers values over arbitrary time intervals and the three gates regulate information flows into and out of the cell. In this paper, we did not use a fully-connected layer to follow this layer, as this will result into worse performance. The number of neurons in this layer was set to 32, so the output size of this layer is 64.

#### Softmax layer

In order to get a probability distribution over two labels which separately represent bound or non-bound sequences, a softmax layer is used in this model. It is composed of two neurons, each of which is densely connected with the previous layer and computes a probability.

### Results merging

MIL is commonly based on an assumption that a bag is labeled as positive if there is at least one instance that contains TFBS, and is labeled as negative if there are no any instances that contain TFBS. Therefore the *Max* function is frequently used as the fusion function in MIL. But *Max* only focuses on the most informative instance and overlooks other instances that may contain useful information. Therefore WSCNN used three additional fusion methods (*Linear Regression*, *Average*, and *Top-Bottom Instances*^33^) to utilize all instances that may contain useful information. However, both *Average* and *Linear Regression* take advantage of all information, inevitably containing useless information, and *Top-Bottom Instances* needs to manually determine the number of the highest and lowest scoring instances. Moreover, how to effectively take advantage of abundant positive instances is also a key point. To solve the above problems, we find a better and more elegant fusion method, named *Noisy-and*^26^, which is based on a different assumption that a bag is labeled as positive if the number of positive instances in the bag exceeds a threshold. This method is defined as follows:

2 $${P}_{i}=\frac{\sigma (a({\bar{p}}_{i}-{b}_{i}))-\sigma (\,-\,a{b}_{i})}{\sigma (a(1-{b}_{i}))-\sigma (\,-\,a{b}_{i})},\,{\bar{p}}_{i}=\tfrac{1}{{n}_{i}}\sum _{j}{p}_{i,j}$$

where *P*_*i*,*j*_ denotes the score of the *j*-th instance at the *i*-th bag, and *n*_*i*_ denotes the number of instances in the *i*-th bag, and ${\bar{P}}_{i}$ denotes the average score over *n* instances in the *i*-th bag. *Noisy-and* is designed to activate a bag level probability *P*_*i*_ once the mean of the instance level probabilities ${\bar{P}}_{i}$ exceeds a certain threshold. *a* is a fixed hyper-parameter that controls the slope of *Noisy-and*. *b*_*i*_ represents an adaptable soft threshold for each class *i* and needs to be learned during training. *σ*(a(1 − *b*_*i*_)) and σ(−*ab*_*i*_) are included to normalized *P*_*i*_ to [0, 1] for *b*_*i*_ in [0, 1] and *a* > 0.

Through this stage, the predicted values of all instances in a bag are fused to yield a final prediction (probability) over ‘bound’ and ‘non-bound’ labels.

**Figure 2:**
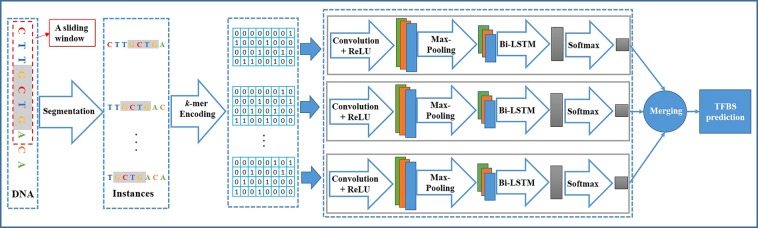
A graphical illustration of the proposed framework.

In summary, the proposed framework is arranged in this order: data processing (segmentation + *k*-mer encoding) → a convolutional layer → a max-pooling layer → a dropout layer → a bi-directional recurrent layer → a dropout layer → a softmax layer → a fusion layer. A graphical illustration of the proposed framework is shown in Fig. 2.

## Results

For brevity, the proposed framework is named as WSCNNLSTM. In this section, the performance of WSCNNLSTM is systematically evaluated by comparing it with other deep-learning based algorithms. We carried out a series of experiments on *in-vivo* ChIP-Seq datasets to show that the overall performance of WSCNNLSTM is superior to the competing methods.

### Experimental setup

#### Data preparation

We collected 50 public ChIP-seq datasets from the HaibTfbs group, which stems from three different cell lines (Gm12878, H1hesc, and K562). For each public dataset, an average number of ~15000 top ranking sequences were chosen as the positive data where each sequence is composed of 200 bps, and the corresponding negative data was generated by matching the repeat fraction, length and GC content of the positive ones following the work^9^, and the number of the negative data is 1~3 times more than the positive data. Moreover, 1/8 of the training data were randomly sampled as the validation data during training.

#### Competing methods

To better evaluate the performance of WSCNNLSTM, we constructed three deep-learning based models, which are similar to DeepBind^10^, DanQ^12^, and WSCNN^20^, respectively.

Model 1: This model is a single-instance learning (SIL) based method, and has the similar architecture to DeepBind. It is arranged in this order: data processing (one-hot encoding) → a convolutional layer → a global max-pooling layer → a fully connected layer → a dropout layer → a softmax layer.

Model 2: This model is a single-instance learning (SIL) based method, and has the similar architecture to DanQ. It is arranged in this order: data processing (one-hot encoding) → a convolutional layer → a max-pooling layer → a dropout layer → a bi-directional recurrent layer → a dropout layer → a softmax layer.

Model 3: This model is a multiple-instance learning (MIL) based method, and has the similar architecture to WSCNN. It is arranged in this order: data processing (segmentation + one-hot encoding) → a convolutional layer → a global max-pooling layer → a fully connected layer → a dropout layer → a softmax layer → a fusion layer.

#### Evaluation metrics

To comprehensively assess the performance of WSCNNLSTM, we adopted three standard evaluation metrics in this paper, including area under receiver operating characteristic curve (ROC AUC), area under precision-recall curve (PR AUC), and F1-score, which are widely used in machine learning and motif discovery^34–46^.

ROC AUC^47^ and PR AUC two commonly-used metrics in which PR AUC is often used under the situation of imbalanced data. Since PR AUC needs not to consider the number of true negative samples, thus it is less prone to influenced by the class imbalance than the ROC AUC metric is^12,48^.

F1-score is a solid metric to measure the classification performance of the classifier, which simultaneously takes into consideration the precision and the recall when computing the score of a test^49^.

#### Hyper-parameter settings

We implemented WSCNNLSTM and the competing methods by Keras with the tensorflow backend, which are freely available at: https://github.com/turningpoint1988/WSCNNLSTM. The parameters in the deep-learning based methods were initialized by Glorot uniform initializer^50^, and optimized by AdaDelta algorithm^51^ with a mini-batchsize of 300. For some sensitive hyper-parameters (i.e., dropout ratio, Momentum in AdaDelta, Delta in AdaDelta), we selected the best configuration using a grid-search strategy. Epochs of training were set to 60, where after each epoch of training, the accuracy of the validation set was assessed and monitored, and the model with the best accuracy in the validation set was saved. The instance length *c* and segmentation stride *s* were set to 120 and 10, since WSCNN has stated that the two hyper-parameters has little effect on the main conclusion. The hyper-parameter *a* in *Noisy-and* was set to 7.5 following the work^26^. The hyper-parameter settings of all the deep-learning based methods in this paper are detailed in Table 1.

**Table 1:** Hyper-parameter Settings.

Methods	Model 1 (DeepBind)	Model 2 (DanQ)	Model 3 (WSCNN)	WSCNNLSTM
Hyper-parameters				
Dropout ratio	0.75, 0.5, 0.1			
Momentum in AdaDelta	0.999, 0.99, 0.9			
Delta in AdaDelta	1e-4, 1e-6, 1e-8			
Learning rate	1	1	1	1
Weight decay	0.0005	0.0005	0.0005	0.0005
Numbers of convolutional neurons	16	16	16	16
Convolutional kernel size	1 × 24	1 × 24	1 × 24	1 × 24
Pooling size	global	1 × 8	global	1 × 8
Numbers of bi-LSTM neurons	—	32	—	32
Neurons (fully-connected layer)	32	—	32	—
Neurons (softmax layer)	2	2	2	2
Epochs	60	60	60	60

### Performance comparison on *in-vivo* data

**Figure 3:**
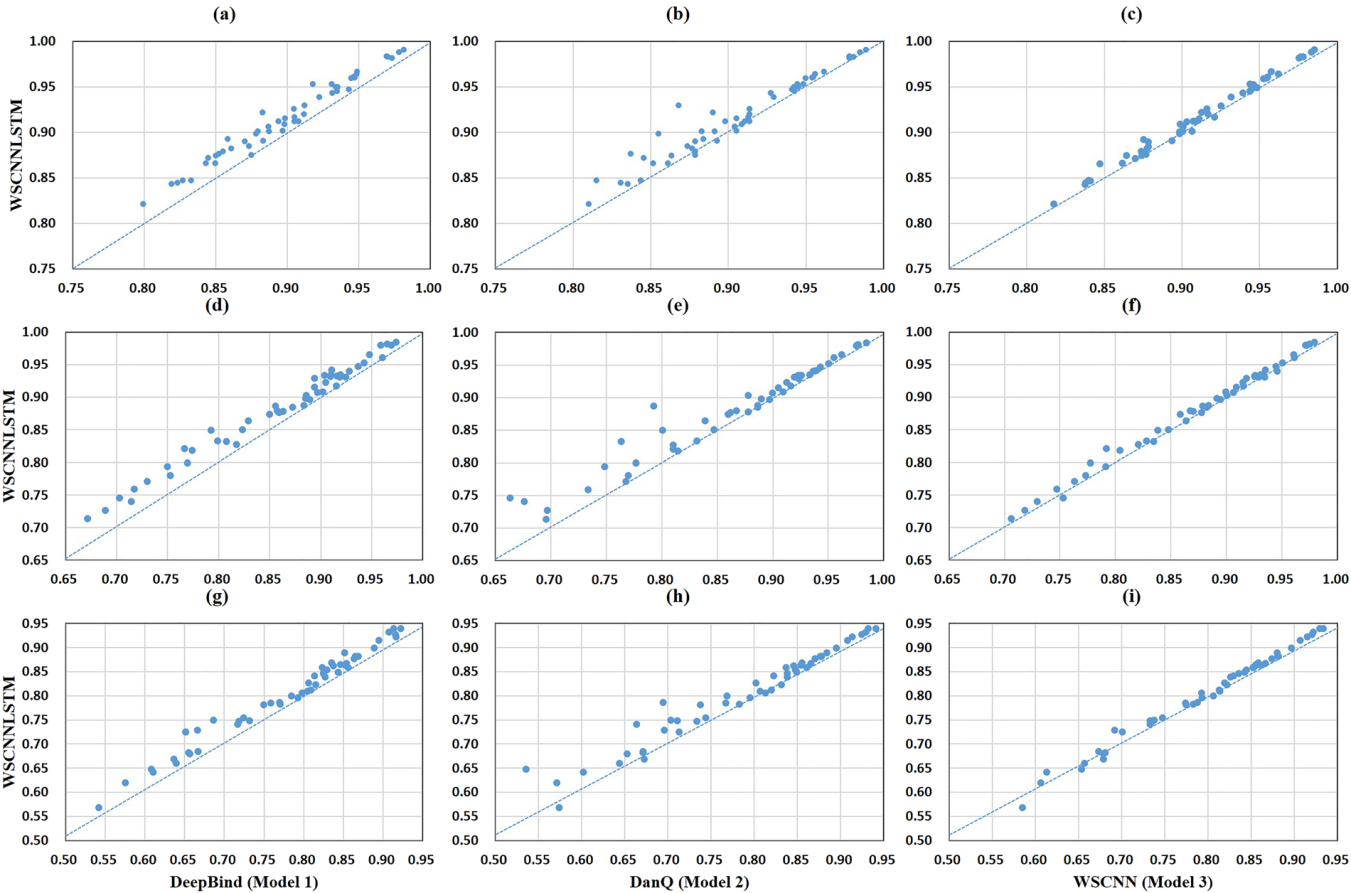
A comparison of WSCNNLSTM and the competing methods on *in-vivo* data, where the first column corresponds to a comparison of WSCNNLSTM and DeepBind under the ROC AUC, PR AUC and F1-score metrics, and the second column corresponds to a comparison of WSCNNLSTM and DanQ, and the third column corresponds to a comparison of WSCNNLSTM and WSCNN.

A comparison of WSCNNLSTM and the competing methods on 50 *in-vivo* ChIP-seq datasets is shown in Fig. 3 and Supplementary Tables 1, 2, 3. Evaluation is done with three-fold cross validation, and prediction performance is measured by the PR AUC, ROC AUC and F1-score metrics.

**Figure 4:**
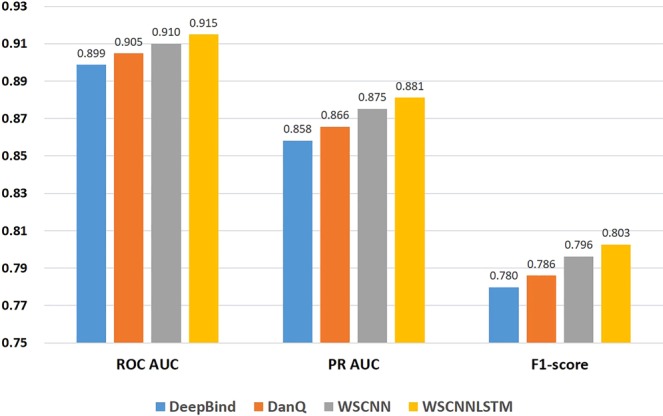
A comparison of WSCNNLSTM and the competing methods on *in-vivo* data under the ROC AUC, PR AUC and F1-score metrics.

Figure 3a,d,g show a superior performance of WSCNNLSTM over DeepBind under the ROC AUC, PR AUC, and F1-score metrics. As WSCNNLSTM combines MIL for learning the weakly supervised information of sequences with RNN for capturing the forward and backward long-term dependencies between the motif features, so it outperforms DeepBind by a large margin. Figure 3b,e,h show a superior performance of WSCNNLSTM over DanQ under the ROC AUC, PR AUC, and F1-score metrics, demonstrating the advantages of allowing for the weakly supervised information of sequences. Figure 3c,e,i show a superior performance of WSCNNLSTM over WSCNN under the ROC AUC, PR AUC, and F1-score metrics, demonstrating the benefits of allowing for the forward and backward long-term dependencies between the motif features. Figure 4 records the average values on ROC AUC, PR AUC, and F1-score, which also shows the consistent conclusion that WSCNNLSTM outperforms the three competing methods. In summary, the above results show that the overall performance of WSCNNLSTM is better than DeepBind, DanQ, and WSCNN.

### The *k*-mer encoding method can significantly improve the performance of modeling *in-vivo* protein-DNA binding

Unlike one-hot encoding, the *k-*mer encoding method can take into consideration the high-order dependencies among nucleotides, which may improve the performance of modeling *in-vivo* protein-DNA binding. As stated by Zhou *et al*.^24^, 2-mer (dinucleotide) and 3-mer (trinucleotide) may contain implicit DNA shape information and partially capture the effect of the DNA shape variation on binding. Moreover, Zhang *et al*.^25^ has stated that the number of learnable parameters will grow exponentially with the increase of *k*. Therefore, in order to make a trade-off between performance and computational complexity, we transformed DNA sequences into matrices that consist of 2-mer or 3-mer by setting *k* to 2 or 3 in *k*-mer encoding.

To test the performance of *k*-mer encoding in the weakly supervised framework, we carried out some comparative experiments on 23 ChIP-seq datasets from the Gm12878 cell line, and the detailed results are shown in Supplementary Tables 4, 5.

**Figure 5:**
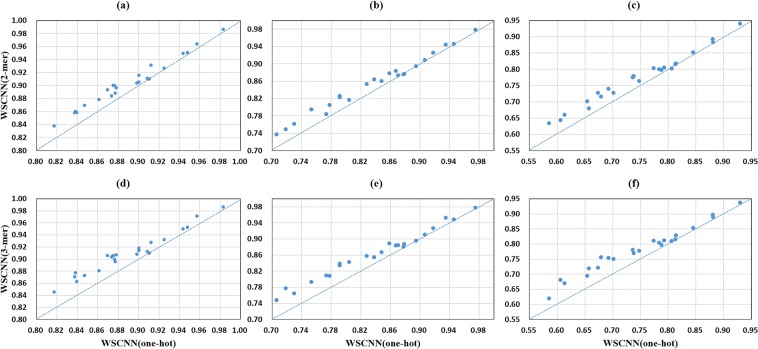
A comparison of WSCNN when using one-hot, 2-mer, and 3-mer encoding, where the first row corresponds to a comparison of WSCNN when using one-hot and 2-mer encoding under the ROC AUC, PR AUC and F1-score metrics, and the second row corresponds to a comparison of WSCNN when using one-hot and 3-mer encoding.

**Figure 6:**
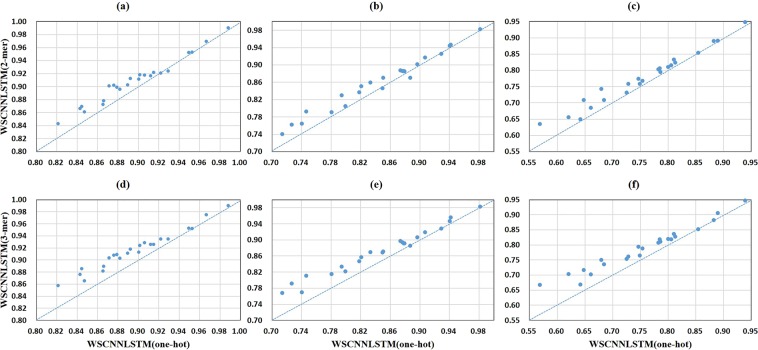
A comparison of WSCNNLSTM when using one-hot, 2-mer, and 3-mer encoding, where the first row corresponds to a comparison of WSCNNLSTM when using one-hot and 2-mer encoding under the ROC AUC, PR AUC and F1-score metrics, and the second row corresponds to a comparison of WSCNNLSTM when using one-hot and 3-mer encoding.

**Figure 7:**
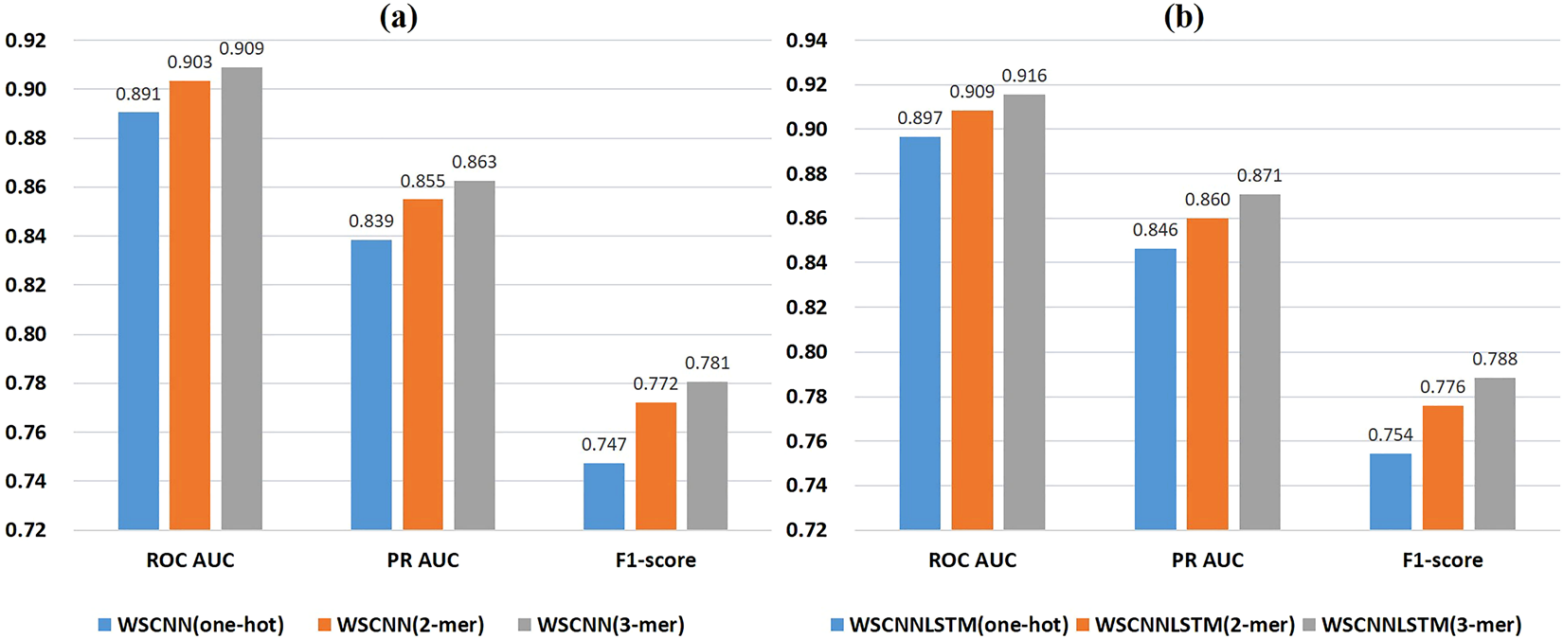
A comparison of WSCNN (**a**) and WSCNNLSTM (**b**) when using one-hot, 2-mer, and 3-mer encoding under the ROC AUC, PR AUC and F1-score metrics.

Figure 5 shows a comparison of one-hot encoding and *k*-mer encoding in WSCNN under the ROC AUC, PR AUC, and F1-score metrics. We can find that the performance of 2-mer and 3-mer encoding is much better than that of one-hot encoding, demonstrating the effectiveness of *k*-mer encoding for modeling *in-vivo* protein-DNA binding. Figure 6 shows a comparison of one-hot encoding and *k*-mer encoding in WSCNNLSTM under the ROC AUC, PR AUC, and F1-score metrics. We can also find the same trend that the performance of 2-mer and 3-mer encoding is much better. Figure 7 records the average values on ROC AUC, PR AUC, and F1-score, which concludes that *k-*mer encoding is superior to one-hot encoding. Moreover, we find that the performance of WSCNN and WSCNNLSTM is improved with the increase of *k*. We think that the reason of the good performance may lie in: it explicitly considers the high-order dependencies among nucleotides (which contains implicit DNA shape information).

### The *Noisy-and* function is a better fusion method in the weakly supervised framework

WSCNN employed four fusion methods (*Max*, *Linear Regression*, *Average* and *Top-Bottom Instances*) to fuse the predicted values of all instances in a bag, and then selected the best one as the final prediction. However, *Max* only focuses on the most informative instance and overlooks other instances that may contain useful information, and both *Average* and *Linear Regression* take advantage of all information, inevitably containing useless information, and *Top-Bottom Instances* needs to manually determine the number of the highest and lowest scoring instances. Moreover, how to effectively take advantage of abundant positive instances is also a key point. Thus we adopt a better and more elegant fusion method, named *Noisy-and*, in this paper.

To test the performance of *Noisy-and* in the weakly supervised framework, we carried out some comparative experiments on 23 ChIP-seq datasets from the Gm12878 cell line, and the detailed results are shown in Supplementary Tables 6, 7.

**Figure 8:**
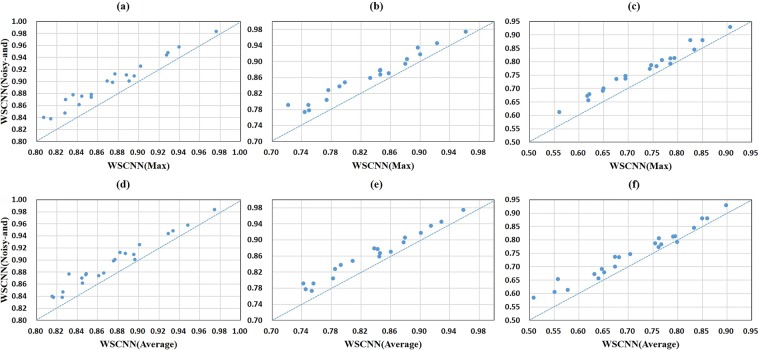
A comparison of WSCNN when using *Max*, *Average*, and *Noisy-and* functions, where the first row corresponds to a comparison of WSCNN when using *Max* and *Noisy-and* under the ROC AUC, PR AUC and F1-score metrics, and the second row corresponds to a comparison of WSCNN when using *Average* and *Noisy-and*.

**Figure 9:**
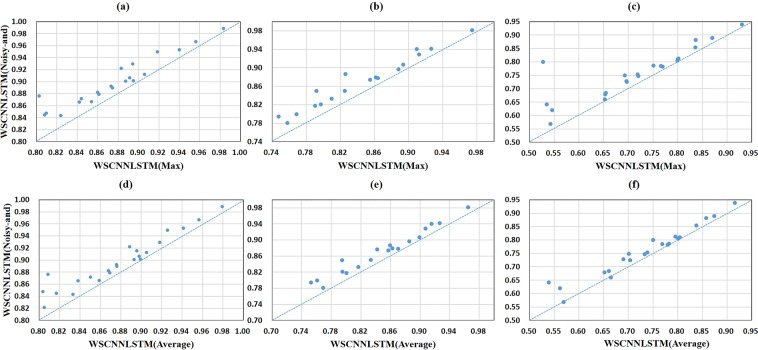
A comparison of WSCNNLSTM when using *Max*, *Average*, and *Noisy-and* functions, where the first row corresponds to a comparison of WSCNNLSTM when using *Max* and *Noisy-and* under the ROC AUC, PR AUC and F1-score metrics, and the second row corresponds to a comparison of WSCNNLSTM when using *Average* and *Noisy-and*.

**Figure 10:**
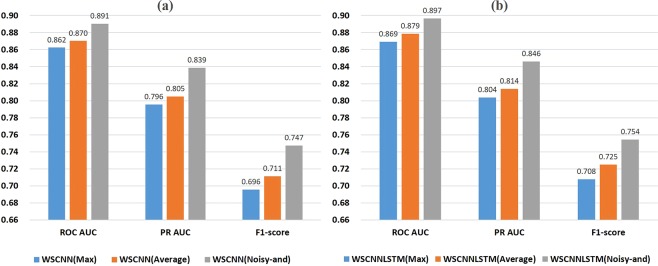
A comparison of WSCNN (**a**) and WSCNNLSTM (**b**) when using *Max*, *Average*, and *Noisy-and* functions under the ROC AUC, PR AUC and F1-score metrics.

Figure 8 shows a comparison of *Noisy-and* and *Max*, *Average* in WSCNN under the ROC AUC, PR AUC, and F1-score metrics. We can find that the performance of *Noisy-and* is much better than that of *Max* and *Average*. Figure 9 shows a comparison of *Noisy-and* and *Max*, *Average* in WSCNNLSTM under the ROC AUC, PR AUC, and F1-score metrics. We find that the performance of *Noisy-and* is also much better than that of *Max* and *Average*. Figure 10 records the average values on ROC AUC, PR AUC, and F1-score, which concludes the same conclusion. We think that the reason of good performance may result from: the weakly supervised framework (WSCNN, WSCNNLSTM) segments DNA sequences into multiple overlapping instances, producing enough positive instances, which is in accordance with the assumption of *Noisy-and* that a bag is labeled as positive if the number of positive instances in the bag exceeds a threshold.

### Adding recurrent layers can improve the performance of the proposed framework

Convolutional layers are often used as motif scanners to capture motifs in the task of modeling *in-vivo* protein-DNA binding, but they ignores the long-term dependencies between motifs. Therefore we add a bi-directional recurrent layer after the convolutional layer in the weakly supervised framework, just like DanQ does, to capture the forward and backward long-term dependencies between motifs.

**Figure 11:**
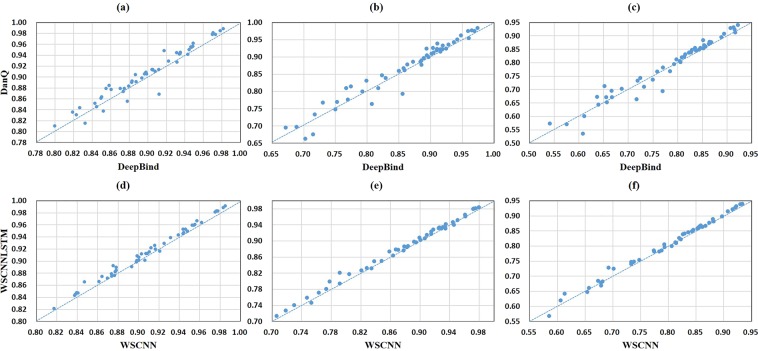
A comparison of models with- and without the recurrent layer, where the first row corresponds to a comparison of DanQ and DeepBind under the ROC AUC, PR AUC and F1-score metrics, and the second row corresponds to a comparison of WSCNNLSTM and WSCNN.

Figure 11 shows a comparison of the methods with RNN (WSCNNLSTM, DanQ) and the ones without RNN (WSCNN, DeepBind) under the ROC AUC, PR AUC and F1-score metrics. The above results show that the models with RNN outperform the ones without RNN by a health margin, demonstrating the effectiveness of allowing for the forward and backward long-term relationship between motif features.

## Conclusions

In this paper, we propose a weakly supervised framework, which combines multiple-instance-learning with a hybrid deep neural network, for modeling *in-vivo* protein-DNA binding. The proposed framework contains three stages: data processing, model designing, and results merging, where the first stage contains the segmentation process and *k*-mer encoding, and the second stage contains a hybrid deep neural network, and the last stage contains the *Noisy-and* fusion method. The experimental results on *in-vivo* ChIP-seq datasets show that our proposed framework performs better than the competing methods. In addition, we also explore the performance of the proposed framework when using *k*-mer encoding and show that the *k*-mer encoding can significantly improve the performance of modeling *in-vivo* protein-DNA binding, and demonstrate that the *Noisy-and* function is a better fusion method in the weakly supervised framework.

From the above results, we find that the performance of WSCNN and WSCNNLSTM is improved with the increase of *k*. However, the big *k* will bring out exponentially growing learnable parameters, and the performance of models may be degenerated when *k* reaches a certain value. Therefore, we will explore the performance of *k*-mer encoding with a big *k* value, and propose a corresponding solution to it in the future works.

## Supplementary information

Supplementary Materials

## Data Availability

The datasets analyzed during the current study are available from the corresponding author on reasonable request.
