# Supplementary material for: Modeling in-vivo protein-DNA binding by combining multiple-instance learning with a hybrid deep neural network
Source: Sci Rep. 2019 Jun 11;9:8484. doi: 10.1038/s41598-019-44966-x (PMC6559991; doi:10.1038/s41598-019-44966-x)
Supplement: Supplementary file 1 — Supplementary Materials [file 41598_2019_44966_MOESM1_ESM.pdf]

Supplementary materials for Modeling *in-vivo* protein-DNA binding by combining multiple-instance learning with a hybrid deep neural network

Qinhu Zhang, Zhen Shen and De-Shuang Huang*

Institute of Machine Learning and Systems Biology, School of Electronics and Information Engineering, Tongji University, Shanghai, 201804, P.R. China

Corresponding author's email: dshuang@tongji.edu.cn

Supplementary Tables

Supplementary Table 1. Cross-validated ROC AUCs of various methods on 50 ChIP-seq datasets, and the best scores are highlighted in bold.

Cell Line	TF	Model 1 Deepbind	Model 2 DanQ	Model 3 WSCNN	WSCNNLSTM	Cell Line	TF	Model 1 Deepbind	Model 2 DanQ	Model 3 WSCNN	WSCNNLSTM
Gm12878	Batf	0.887	0.904	0.901	**0.906**	H1hesc	Rad	0.973	0.978	0.975	**0.981**
Gm12878	Bcl1	0.823	0.831	0.838	**0.844**	H1hesc	Sin3	0.894	0.898	0.903	**0.912**
Gm12878	Bcl3	0.858	0.884	0.875	**0.892**	H1hesc	Sp1	0.874	0.874	0.878	**0.885**
Gm12878	Bclaf	0.843	0.852	0.847	**0.866**	H1hesc	Srf	0.949	0.956	0.962	**0.964**
Gm12878	Ebf	0.861	0.877	0.877	**0.882**	H1hesc	Taf1	0.908	0.911	0.907	**0.912**
Gm12878	Egr1	0.934	0.942	0.948	**0.949**	H1hesc	Tcf12	0.850	0.863	0.864	**0.874**
Gm12878	Elf1	0.905	**0.914**	0.909	0.912	H1hesc	Usf1	0.970	0.978	0.977	**0.983**
Gm12878	Ets1	0.912	0.868	0.925	**0.929**	H1hesc	Yy1	0.923	0.929	0.931	**0.939**
Gm12878	Irf4	0.833	0.815	0.840	**0.847**	K562	Atf3	0.931	0.945	0.946	**0.952**
Gm12878	Mef2a	0.852	0.837	**0.877**	0.876	K562	E2f6	0.935	0.943	0.944	**0.945**
Gm12878	Nrsf	0.899	0.905	0.911	**0.915**	K562	Egr1	0.947	0.954	0.955	**0.960**
Gm12878	Pax5c20	0.850	0.861	0.862	**0.866**	K562	Elf1	0.943	0.941	0.944	**0.947**
Gm12878	Pax5n19	0.845	0.845	0.870	**0.872**	K562	Ets1	0.883	0.893	**0.894**	0.891
Gm12878	Pbx3	0.855	0.879	0.874	**0.879**	K562	Fosl1	0.935	0.945	0.948	**0.949**
Gm12878	Pou2	0.819	0.835	0.838	**0.843**	K562	Gabp	0.932	0.927	0.939	**0.943**
Gm12878	Pu1	0.949	0.962	0.957	**0.967**	K562	Gata2	0.827	0.844	0.841	**0.847**
Gm12878	Rad21	0.978	0.985	0.983	**0.988**	K562	Hey1	0.875	**0.879**	0.874	0.875
Gm12878	Sp1	0.800	0.810	0.817	**0.821**	K562	Max	0.905	0.914	0.916	**0.926**
Gm12878	Srf	0.883	0.890	0.912	**0.922**	K562	Nrsf	0.880	0.883	**0.906**	0.901
Gm12878	Taf1	0.897	**0.905**	0.900	0.901	K562	Pu1	0.971	0.981	0.978	**0.983**
Gm12878	Tcf12	0.871	0.879	0.878	**0.890**	K562	Rad21	0.982	0.989	0.985	**0.991**
Gm12878	Usf1	0.918	0.948	0.944	**0.953**	K562	Srf	0.878	0.855	0.898	**0.898**
Gm12878	Yy1	0.888	0.891	0.898	**0.901**	K562	Taf1	0.898	0.909	0.899	**0.909**
H1hesc	Gabp	0.905	0.913	**0.921**	0.916	K562	Usf1	0.947	0.955	0.955	**0.961**
H1hesc	Nrsf	0.945	0.950	0.953	**0.959**	K562	Yy1	0.912	0.914	0.917	**0.920**
						**Average ROC AUC**		0.899	0.905	0.910	**0.915**

**P-value**	WSCNNLSTM vs Deepbind<br>p-value < 2.2e-16	WSCNNLSTM vs DanQ<br>p-value < 6.278e-07	WSCNNLSTM vs WSCNN<br>p-value ⩽ 3.106e-10

Supplementary Table 2. Cross-validated PR AUCs of various methods on 50 ChIP-seq datasets, and the best scores are highlighted in bold.

Cell Line	TF	Model 1 Deepbind	Model 2 DanQ	Model 3 WSCNN	WSCNNLSTM	Cell Line	TF	Model 1 Deepbind	Model 2 DanQ	Model 3 WSCNN	WSCNNLSTM
Gm12878	Batf	0.799	0.832	0.828	**0.833**	H1hesc	Rad21	0.969	0.976	0.973	**0.980**
Gm12878	Bcl1	0.688	0.697	0.718	**0.727**	H1hesc	Sin3	0.885	0.890	0.892	**0.898**
Gm12878	Bcl3	0.767	0.810	0.792	**0.821**	H1hesc	Sp1	0.818	0.810	0.821	**0.827**
Gm12878	Bclaf	0.770	0.777	0.778	**0.799**	H1hesc	Srf	0.910	0.923	0.931	**0.934**
Gm12878	Ebf	0.823	0.847	0.848	**0.850**	H1hesc	Taf1	0.915	0.916	0.915	**0.917**
Gm12878	Egr1	0.928	0.936	**0.946**	0.940	H1hesc	Tcf12	0.717	0.734	0.747	**0.759**
Gm12878	Elf1	0.889	0.897	0.894	**0.897**	H1hesc	Usf1	0.948	0.962	0.961	**0.966**
Gm12878	Ets1	0.856	0.793	0.879	**0.887**	H1hesc	Yy1	0.905	0.913	0.915	**0.923**
Gm12878	Irf4	0.715	0.676	0.730	**0.740**	K562	Atf3	0.904	0.926	0.926	**0.934**
Gm12878	Mef2a	0.702	0.663	**0.753**	0.746	K562	E2f6	0.937	0.943	0.945	**0.947**
Gm12878	Nrsf	0.859	0.862	**0.878**	0.877	K562	Egr1	0.942	0.950	0.951	**0.952**
Gm12878	Pax5c20	0.857	0.867	0.867	**0.879**	K562	Elf1	0.960	0.955	0.961	**0.961**
Gm12878	Pax5n19	0.750	0.748	0.791	**0.794**	K562	Ets1	0.872	**0.887**	0.882	0.885
Gm12878	Pbx3	0.774	0.815	0.804	**0.818**	K562	Fosl1	0.894	0.905	0.909	**0.915**
Gm12878	Pou2	0.753	0.770	0.773	**0.781**	K562	Gabp	0.886	0.878	0.901	**0.903**
Gm12878	Pu1	0.911	0.939	0.935	**0.942**	K562	Gata2	0.730	0.768	0.763	**0.771**
Gm12878	Rad21	0.965	0.977	0.975	**0.982**	K562	Hey1	0.884	0.886	0.884	**0.888**
Gm12878	Sp1	0.671	0.696	0.706	**0.714**	K562	Max	0.916	0.923	0.926	**0.933**
Gm12878	Srf	0.793	0.800	0.838	**0.850**	K562	Nrsf	0.829	0.839	0.864	**0.864**
Gm12878	Taf1	0.863	0.878	0.870	**0.878**	K562	Pu1	0.959	0.975	0.972	**0.980**
Gm12878	Tcf12	0.850	0.860	0.859	**0.874**	K562	Rad21	0.974	0.984	0.979	**0.984**
Gm12878	Usf1	0.894	0.924	0.918	**0.929**	K562	Srf	0.807	0.764	**0.834**	0.832
Gm12878	Yy1	0.897	0.900	0.906	**0.907**	K562	Taf1	0.902	**0.910**	0.899	0.909
H1hesc	Gabp	0.919	0.923	**0.934**	0.931	K562	Usf1	0.919	0.933	0.933	**0.935**
H1hesc	Nrsf	0.910	0.919	0.925	**0.932**	K562	Yy1	0.924	0.924	0.929	**0.931**
						**Average PR AUC**		0.858	0.866	0.875	**0.881**

**P-value**	WSCNNLSTM vs Deepbind	WSCNNLSTM vs DanQ	WSCNNLSTM vs WSCNN
	p-value < 6.03e-16	p-value < 5.74e-06	p-value < 1.318e-08

Supplementary Table 3. Cross-validated F1-scores of various methods on 50 ChIP-seq datasets, and the best scores are highlighted in bold.

Cell Line	TF	Model 1 Deepbind	Model 2 DanQ	Model 3 WSCNN	WSCNNLSTM	Cell Line	TF	Model 1 Deepbind	Model 2 DanQ	Model 3 WSCNN	WSCNNLSTM
Gm12878	Batf	0.719	0.734	0.736	**0.747**	H1hesc	Rad21	0.915	0.925	0.921	**0.928**
Gm12878	Bcl1	0.575	0.571	0.606	**0.620**	H1hesc	Sin3	0.814	0.822	0.829	**0.841**
Gm12878	Bcl3	0.666	0.695	0.692	**0.728**	H1hesc	Sp1	0.732	0.711	0.733	**0.748**
Gm12878	Bclaf	0.667	0.671	0.673	**0.685**	H1hesc	Srf	0.823	0.837	0.852	**0.858**
Gm12878	Ebf	0.724	0.744	0.748	**0.754**	H1hesc	Taf1	0.843	0.849	0.843	**0.849**
Gm12878	Egr1	0.865	0.878	0.881	**0.882**	H1hesc	Tcf12	0.655	0.671	0.681	**0.682**
Gm12878	Elf1	0.809	**0.820**	0.813	0.812	H1hesc	Usf1	0.894	0.908	0.907	**0.915**
Gm12878	Ets1	0.770	0.695	**0.788**	0.786	H1hesc	Yy1	0.825	0.838	0.836	**0.846**
Gm12878	Irf4	0.611	0.602	0.613	**0.642**	K562	Atf3	0.835	0.855	0.858	**0.868**
Gm12878	Mef2a	0.608	0.535	**0.654**	0.648	K562	E2f6	0.868	0.877	0.881	**0.882**
Gm12878	Nrsf	0.784	0.768	**0.806**	0.800	K562	Egr1	0.888	0.895	0.896	**0.899**
Gm12878	Pax5c20	0.770	0.783	**0.783**	0.782	K562	Elf1	0.916	0.913	0.916	**0.922**
Gm12878	Pax5n19	0.657	0.653	0.679	**0.680**	K562	Ets1	0.815	**0.832**	0.822	0.823
Gm12878	Pbx3	0.651	0.713	0.701	**0.725**	K562	Fosl1	0.856	**0.861**	0.853	0.859
Gm12878	Pou2	0.640	0.643	0.657	**0.661**	K562	Gabp	0.806	0.802	0.819	**0.827**
Gm12878	Pu1	0.852	0.884	0.880	**0.890**	K562	Gata2	0.637	0.673	**0.679**	0.668
Gm12878	Rad21	0.913	0.932	0.930	**0.939**	K562	Hey1	0.793	0.795	0.793	**0.796**
Gm12878	Sp1	0.541	0.574	**0.585**	0.568	K562	Max	0.846	0.855	0.856	**0.865**
Gm12878	Srf	0.686	0.703	0.738	**0.749**	K562	Nrsf	0.750	0.737	0.775	**0.782**
Gm12878	Taf1	0.798	**0.813**	0.793	0.806	K562	Pu1	0.907	0.929	0.922	**0.932**
Gm12878	Tcf12	0.759	0.767	0.774	**0.785**	K562	Rad21	0.922	**0.941**	0.933	0.940
Gm12878	Usf1	0.829	0.848	0.845	**0.854**	K562	Srf	0.717	0.664	0.733	**0.741**
Gm12878	Yy1	0.804	0.806	**0.814**	0.810	K562	Taf1	0.827	0.839	0.826	**0.839**
H1hesc	Gabp	0.864	0.870	0.874	**0.877**	K562	Usf1	0.854	0.866	0.867	**0.867**
H1hesc	Nrsf	0.837	0.846	0.855	**0.862**	K562	Yy1	0.852	0.854	0.861	**0.864**
						**Average F1-score**		0.780	0.786	0.796	**0.803**

	WSCNNLSTM vs Deepbind	WSCNNLSTM vs DanQ	WSCNNLSTM vs WSCNN
**P-value**	p-value < 1.469e-14	p-value < 1.438e-05	p-value < 4.141e-06

Supplementary Table 4. We compare the performance of WSCNN when mononucleotide (1-mer), dinucleotide (2-mer), and trinucleotide (3-mer) are used respectively.

Supplementary Table 4.1: ROC AUC of WSCNN on the 23 ChIP-seq datasets in Gm12878 cell line, and the best scores are highlighted in bold.

Cell Line	TF	WSCNN			Cell Line	TF	WSCNN		
		1-mer	2-mer	3-mer			1-mer	2-mer	3-mer
Gm12878	Batf	0.901	0.916	**0.918**	Gm12878	Pax5n19	0.870	0.893	**0.905**
Gm12878	Bcl1	0.838	0.860	**0.877**	Gm12878	Pbx3	0.874	0.884	**0.904**
Gm12878	Bcl3	0.875	0.900	**0.906**	Gm12878	Pou2	0.838	0.858	**0.870**
Gm12878	Bclaf	0.847	0.869	**0.873**	Gm12878	Pu1	0.957	0.964	**0.971**
Gm12878	Ebf	0.877	0.888	**0.896**	Gm12878	Rad21	0.983	0.986	**0.986**
Gm12878	Egr1	0.948	0.950	**0.953**	Gm12878	Sp1	0.817	0.838	**0.845**
Gm12878	Elf1	0.909	0.911	**0.912**	Gm12878	Srf	0.912	**0.931**	0.928
Gm12878	Ets1	0.925	0.926	**0.932**	Gm12878	Taf1	0.900	0.905	**0.914**
Gm12878	Irf4	0.840	0.859	**0.862**	Gm12878	Tcf12	0.878	0.896	**0.907**
Gm12878	Mef2a	0.877	0.900	**0.900**	Gm12878	Usf1	0.944	0.949	**0.950**
Gm12878	Nrsf	**0.911**	0.910	0.910	Gm12878	Yy1	0.898	0.904	**0.908**
Gm12878	Pax5c20	0.862	0.878	**0.880**	**Average ROC AUC**		0.891	0.903	**0.909**
**P-value**		2-mer encoding vs one-hot encoding					3-mer encoding vs one-hot encoding		
		p-value ⩽ 3.954e-07					p-value ⩽ 1.041e-07		

Supplementary Table 4.2: PR AUC of WSCNN on the 23 ChIP-seq datasets in Gm12878 cell line, and the best scores are highlighted in bold.

Cell Line	TF	WSCNN			Cell Line	TF	WSCNN		
		1-mer	2-mer	3-mer			1-mer	2-mer	3-mer
Gm12878	Batf	0.828	0.853	**0.858**	Gm12878	Pax5n19	0.791	0.823	**0.839**
Gm12878	Bcl1	0.718	0.749	**0.777**	Gm12878	Pbx3	0.804	0.817	**0.843**
Gm12878	Bcl3	0.792	0.826	**0.835**	Gm12878	Pou2	0.773	0.784	**0.809**
Gm12878	Bclaf	0.778	0.805	**0.808**	Gm12878	Pu1	0.935	0.944	**0.952**
Gm12878	Ebf	0.848	0.861	**0.867**	Gm12878	Rad21	0.975	**0.979**	0.978
Gm12878	Egr1	0.946	0.946	**0.949**	Gm12878	Sp1	0.706	0.738	**0.748**
Gm12878	Elf1	0.894	0.895	**0.896**	Gm12878	Srf	0.838	**0.864**	0.854
Gm12878	Ets1	0.879	0.877	**0.888**	Gm12878	Taf1	0.870	0.874	**0.884**
Gm12878	Irf4	0.730	0.762	**0.765**	Gm12878	Tcf12	0.859	0.878	**0.889**
Gm12878	Mef2a	0.753	**0.795**	0.794	Gm12878	Usf1	0.918	0.926	**0.927**
Gm12878	Nrsf	0.878	0.876	**0.881**	Gm12878	Yy1	0.906	0.909	**0.911**
Gm12878	Pax5c20	0.867	0.884	**0.884**	**Average PR AUC**		0.839	0.855	**0.863**
**P-value**		2-mer encoding vs one-hot encoding					3-mer encoding vs one-hot encoding		
		p-value ⩽ 7.247e-06					p-value ⩽ 8.034e-07		

Supplementary Table 4.3: F1-scores of WSCNN on the 23 ChIP-seq datasets in Gm12878 cell line, and the best scores are highlighted in bold.

Cell Line	TF	WSCNN			Cell Line	TF	WSCNN		
		1-mer	2-mer	3-mer			1-mer	2-mer	3-mer
Gm12878	Batf	0.736	0.776	**0.781**	Gm12878	Pax5n19	0.679	0.716	**0.756**
Gm12878	Bcl1	0.606	0.643	**0.682**	Gm12878	Pbx3	0.701	0.728	**0.751**
Gm12878	Bcl3	0.692	0.740	**0.754**	Gm12878	Pou2	0.657	0.680	**0.720**
Gm12878	Bclaf	0.673	**0.728**	0.722	Gm12878	Pu1	0.880	0.892	**0.898**
Gm12878	Ebf	0.748	0.763	**0.778**	Gm12878	Rad21	0.930	**0.940**	0.938
Gm12878	Egr1	0.881	0.883	**0.889**	Gm12878	Sp1	0.585	**0.635**	0.620
Gm12878	Elf1	0.813	0.815	**0.815**	Gm12878	Srf	0.738	**0.779**	0.770
Gm12878	Ets1	0.788	0.798	**0.798**	Gm12878	Taf1	0.793	0.806	**0.812**
Gm12878	Irf4	0.613	0.660	**0.670**	Gm12878	Tcf12	0.774	0.803	**0.811**
Gm12878	Mef2a	0.654	**0.701**	0.695	Gm12878	Usf1	0.845	0.852	**0.854**
Gm12878	Nrsf	0.806	0.802	**0.810**	Gm12878	Yy1	0.814	0.818	**0.829**
Gm12878	Pax5c20	0.783	0.800	**0.804**	**Average F1-score**		0.747	0.772	**0.781**
**P-value**	2-mer encoding vs one-hot encoding<br>p-value $\leqslant$ 1.661e-06					3-mer encoding vs one-hot encoding<br>p-value $\leqslant$ 6.595e-07			

Supplementary Table 5. We compare the performance of WSCNNLSTM when mononucleotide (1-mer), dinucleotide (2-mer), and trinucleotide (3-mer) are used respectively.

Supplementary Table 5.1: ROC AUC of WSCNNLSTM on the 23 ChIP-seq datasets in Gm12878 cell line, and the best scores are highlighted in bold.

Cell Line	TF	WSCNNLSTM			Cell Line	TF	WSCNNLSTM		
		1-mer	2-mer	3-mer			1-mer	2-mer	3-mer
Gm12878	Batf	0.906	0.917	**0.928**	Gm12878	Pax5n19	0.872	0.901	**0.903**
Gm12878	Bcl1	0.844	0.869	**0.885**	Gm12878	Pbx3	0.879	0.899	**0.909**
Gm12878	Bcl3	0.892	0.912	**0.918**	Gm12878	Pou2	0.843	0.866	**0.876**
Gm12878	Bclaf	0.866	0.872	**0.881**	Gm12878	Pu1	0.967	0.969	**0.975**
Gm12878	Ebf	0.882	0.896	**0.903**	Gm12878	Rad21	0.988	0.990	**0.990**
Gm12878	Egr1	0.949	0.952	**0.952**	Gm12878	Sp1	0.821	0.843	**0.857**
Gm12878	Elf1	0.912	0.916	**0.925**	Gm12878	Srf	0.922	0.921	**0.935**
Gm12878	Ets1	0.929	0.924	**0.935**	Gm12878	Taf1	0.901	0.918	**0.924**
Gm12878	Irf4	0.847	0.861	**0.865**	Gm12878	Tcf12	0.890	0.903	**0.911**
Gm12878	Mef2a	0.876	0.902	**0.908**	Gm12878	Usf1	0.953	**0.953**	0.952
Gm12878	Nrsf	0.915	0.922	**0.926**	Gm12878	Yy1	0.901	0.911	**0.913**
Gm12878	Pax5c20	0.866	0.878	**0.889**	**Average ROC AUC**		0.897	0.909	**0.916**
**P-value**	2-mer encoding vs one-hot encoding<br>p-value ⩽ 5.89e-06				3-mer encoding vs one-hot encoding<br>p-value ⩽ 6.953e-08				

Supplementary Table 5.2: PR AUC of WSCNNLSTM on the 23 ChIP-seq datasets in Gm12878 cell line, and the best scores are highlighted in bold.

Cell Line	TF	WSCNNLSTM			Cell Line	TF	WSCNNLSTM		
		1-mer	2-mer	3-mer			1-mer	2-mer	3-mer
Gm12878	Batf	0.833	0.860	**0.870**	Gm12878	Pax5n19	0.794	0.830	**0.834**
Gm12878	Bcl1	0.727	0.762	**0.792**	Gm12878	Pbx3	0.818	0.837	**0.847**
Gm12878	Bcl3	0.821	0.851	**0.857**	Gm12878	Pou2	0.781	0.791	**0.815**
Gm12878	Bclaf	0.799	0.805	**0.822**	Gm12878	Pu1	0.942	0.947	**0.956**
Gm12878	Ebf	0.850	0.870	**0.872**	Gm12878	Rad21	0.982	0.982	**0.983**
Gm12878	Egr1	0.940	0.944	**0.947**	Gm12878	Sp1	0.714	0.741	**0.768**
Gm12878	Elf1	0.897	0.901	**0.906**	Gm12878	Srf	0.850	0.846	**0.869**
Gm12878	Ets1	**0.887**	0.870	0.886	Gm12878	Taf1	0.878	0.886	**0.891**
Gm12878	Irf4	0.740	0.764	**0.770**	Gm12878	Tcf12	0.874	0.887	**0.897**
Gm12878	Mef2a	0.746	0.792	**0.811**	Gm12878	Usf1	**0.929**	0.926	0.928
Gm12878	Nrsf	0.877	0.886	**0.894**	Gm12878	Yy1	0.907	0.917	**0.919**
Gm12878	Pax5c20	0.879	0.885	**0.892**	**Average PR AUC**		0.846	0.860	**0.871**
**P-value**	2-mer encoding vs one-hot encoding<br>p-value ⩽ 2.457e-04				3-mer encoding vs one-hot encoding<br>p-value ⩽ 3.109e-06				

Supplementary Table 5.3: F1-score of WSCNNLSTM on the 23 ChIP-seq datasets in Gm12878 cell line, and the best scores are highlighted in bold.

Cell Line	TF	WSCNNLSTM			Cell Line	TF	WSCNNLSTM		
		1-mer	2-mer	3-mer			1-mer	2-mer	3-mer
Gm12878	Batf	0.747	0.774	**0.794**	Gm12878	Pax5n19	0.680	0.743	**0.750**
Gm12878	Bcl1	0.620	0.656	**0.704**	Gm12878	Pbx3	0.725	0.732	**0.753**
Gm12878	Bcl3	0.728	0.758	**0.761**	Gm12878	Pou2	0.661	0.685	**0.702**
Gm12878	Bclaf	0.685	0.709	**0.735**	Gm12878	Pu1	0.890	0.891	**0.906**
Gm12878	Ebf	0.754	0.768	**0.788**	Gm12878	Rad21	0.939	**0.948**	0.947
Gm12878	Egr1	0.882	**0.890**	0.883	Gm12878	Sp1	0.568	0.635	**0.667**
Gm12878	Elf1	0.812	0.824	**0.828**	Gm12878	Srf	0.749	0.759	**0.765**
Gm12878	Ets1	0.786	0.795	**0.811**	Gm12878	Taf1	0.806	0.816	**0.819**
Gm12878	Irf4	0.642	0.649	**0.668**	Gm12878	Tcf12	0.785	0.807	**0.819**
Gm12878	Mef2a	0.648	0.709	**0.717**	Gm12878	Usf1	0.854	**0.854**	0.852
Gm12878	Nrsf	0.800	0.811	**0.820**	Gm12878	Yy1	0.810	0.833	**0.836**
Gm12878	Pax5c20	0.782	0.803	**0.807**	**Average F1-score**		0.754	0.776	**0.788**
**P-value**	2-mer encoding vs one-hot encoding				3-mer encoding vs one-hot encoding				
	p-value $\leqslant$ 1.901e-05				p-value $\leqslant$ 2.547e-06				

Supplementary Table 6. We compare the *Noisy-And* function with the other fusion methods (Max and Average) in the framework of WSCNN.

Supplementary Table 6.1: ROC AUC of WSCNN on the 23 ChIP-seq datasets in Gm12878 cell line, and the best scores are highlighted in bold.

Cell Line	TF	WSCNN			Cell Line	TF	WSCNN		
		Max	Average	Noisy-and			Max	Average	Noisy-and
Gm12878	Batf	0.869	0.877	**0.901**	Gm12878	Pax5n19	0.828	0.844	**0.870**
Gm12878	Bcl1	0.787	0.816	**0.838**	Gm12878	Pbx3	0.854	0.861	**0.874**
Gm12878	Bcl3	0.845	0.848	**0.875**	Gm12878	Pou2	0.814	0.825	**0.838**
Gm12878	Bclaf	0.828	0.826	**0.847**	Gm12878	Pu1	0.940	0.949	**0.957**
Gm12878	Ebf	0.836	0.849	**0.877**	Gm12878	Rad21	0.976	0.975	**0.983**
Gm12878	Egr1	0.929	0.934	**0.948**	Gm12878	Sp1	0.776	0.785	**0.817**
Gm12878	Elf1	0.896	0.896	**0.909**	Gm12878	Srf	0.877	0.882	**0.912**
Gm12878	Ets1	0.902	0.901	**0.925**	Gm12878	Taf1	0.891	0.897	**0.900**
Gm12878	Irf4	0.807	0.815	**0.840**	Gm12878	Tcf12	0.854	0.866	**0.878**
Gm12878	Mef2a	0.793	0.832	**0.877**	Gm12878	Usf1	0.927	0.929	**0.944**
Gm12878	Nrsf	0.888	0.887	**0.911**	Gm12878	Yy1	0.875	0.876	**0.898**
Gm12878	Pax5c20	0.842	0.845	**0.862**	**Average ROC AUC**		0.862	0.870	**0.891**
**P-value**		Noisy-and vs Max					Noisy-and vs Average		
		p-value ⩽ 3.929e-08					p-value ⩽ 6.983e-10		

Supplementary Table 6.2: PR AUC of WSCNN on the 23 ChIP-seq datasets in Gm12878 cell line, and the best scores are highlighted in bold.

Cell Line	TF	WSCNN			Cell Line	TF	WSCNN		
		Max	Average	Noisy-and			Max	Average	Noisy-and
Gm12878	Batf	0.776	0.785	**0.828**	Gm12878	Pax5n19	0.722	0.742	**0.791**
Gm12878	Bcl1	0.616	0.664	**0.718**	Gm12878	Pbx3	0.774	0.783	**0.804**
Gm12878	Bcl3	0.749	0.756	**0.792**	Gm12878	Pou2	0.744	0.753	**0.773**
Gm12878	Bclaf	0.750	0.745	**0.778**	Gm12878	Pu1	0.897	0.915	**0.935**
Gm12878	Ebf	0.798	0.809	**0.848**	Gm12878	Rad21	0.962	0.958	**0.975**
Gm12878	Egr1	0.923	0.929	**0.946**	Gm12878	Sp1	0.642	0.653	**0.706**
Gm12878	Elf1	0.880	0.878	**0.894**	Gm12878	Srf	0.791	0.793	**0.838**
Gm12878	Ets1	0.846	0.838	**0.879**	Gm12878	Taf1	0.858	0.860	**0.870**
Gm12878	Irf4	0.661	0.669	**0.730**	Gm12878	Tcf12	0.833	0.845	**0.859**
Gm12878	Mef2a	0.601	0.668	**0.753**	Gm12878	Usf1	0.900	0.901	**0.918**
Gm12878	Nrsf	0.846	0.843	**0.878**	Gm12878	Yy1	0.882	0.880	**0.906**
Gm12878	Pax5c20	0.846	0.846	**0.867**	**Average PR AUC**		0.796	0.805	**0.839**
		Noisy-and vs Max					Noisy-and vs Average		
**P-value**		p-value ⩽ 2.034e-06					p-value ⩽ 1.434e-08		

Supplementary Table 6.3: F1-score of WSCNN on the 23 ChIP-seq datasets in Gm12878 cell line, and the best scores are highlighted in bold.

Cell Line	TF	WSCNN			Cell Line	TF	WSCNN		
		Max	Average	Noisy-and			Max	Average	Noisy-and
Gm12878	Batf	0.675	0.681	**0.736**	Gm12878	Pax5n19	0.621	0.651	**0.679**
Gm12878	Bcl1	0.476	0.550	**0.606**	Gm12878	Pbx3	0.650	0.673	**0.701**
Gm12878	Bcl3	0.648	0.647	**0.692**	Gm12878	Pou2	0.619	0.639	**0.657**
Gm12878	Bclaf	0.617	0.631	**0.673**	Gm12878	Pu1	0.826	0.850	**0.880**
Gm12878	Ebf	0.694	0.704	**0.748**	Gm12878	Rad21	0.906	0.898	**0.930**
Gm12878	Egr1	0.850	0.860	**0.881**	Gm12878	Sp1	0.486	0.508	**0.585**
Gm12878	Elf1	0.785	0.791	**0.813**	Gm12878	Srf	0.694	0.673	**0.738**
Gm12878	Ets1	0.747	0.754	**0.788**	Gm12878	Taf1	0.785	0.799	**0.793**
Gm12878	Irf4	0.560	0.577	**0.613**	Gm12878	Tcf12	0.743	0.761	**0.774**
Gm12878	Mef2a	0.462	0.557	**0.654**	Gm12878	Usf1	0.834	0.833	**0.845**
Gm12878	Nrsf	0.768	0.762	**0.806**	Gm12878	Yy1	0.794	0.795	**0.814**
Gm12878	Pax5c20	0.757	0.767	**0.783**	**Average F1-score**		0.696	0.711	**0.747**
		Noisy-and vs Max					Noisy-and vs Average		
**P-value**		p-value ⩽ 3.65e-06					p-value ⩽ 1.787e-07		

Supplementary Table 7. We compare the *Noisy-And* function with the other fusion methods (Max and Average) in the framework of WSCNNLSTM.

Supplementary Table 7.1: ROC AUC of WSCNNLSTM on the 23 ChIP-seq datasets in Gm12878 cell line, and the best scores are highlighted in bold.

Cell Line	TF	WSCNNLSTM			Cell Line	TF	WSCNNLSTM		
		Max	Average	Noisy-and			Max	Average	Noisy-and
Gm12878	Batf	0.891	0.898	**0.906**	Gm12878	Pax5n19	0.844	0.850	**0.872**
Gm12878	Bcl1	0.808	0.817	**0.844**	Gm12878	Pbx3	0.861	0.869	**0.879**
Gm12878	Bcl3	0.873	0.876	**0.892**	Gm12878	Pou2	0.824	0.833	**0.843**
Gm12878	Bclaf	0.842	0.838	**0.866**	Gm12878	Pu1	0.956	0.957	**0.967**
Gm12878	Ebf	0.860	0.868	**0.882**	Gm12878	Rad21	0.983	0.979	**0.988**
Gm12878	Egr1	0.919	0.926	**0.949**	Gm12878	Sp1	0.795	0.805	**0.821**
Gm12878	Elf1	0.906	0.905	**0.912**	Gm12878	Srf	0.883	0.889	**0.922**
Gm12878	Ets1	0.894	0.918	**0.929**	Gm12878	Taf1	0.895	0.899	**0.901**
Gm12878	Irf4	0.810	0.804	**0.847**	Gm12878	Tcf12	0.875	0.876	**0.890**
Gm12878	Mef2a	0.803	0.808	**0.876**	Gm12878	Usf1	0.940	0.942	**0.953**
Gm12878	Nrsf	0.784	0.896	**0.915**	Gm12878	Yy1	0.888	0.893	**0.901**
Gm12878	Pax5c20	0.854	0.859	**0.866**	**Average ROC AUC**		0.869	0.879	**0.897**
		Noisy-and vs Max					Noisy-and vs Average		
**P-value**		p-value $\leqslant$ 7.052e-05					p-value $\leqslant$ 5.527e-06		

Supplementary Table 7.2: PR AUC of WSCNNLSTM on the 23 ChIP-seq datasets in Gm12878 cell line, and the best scores are highlighted in bold.

Cell Line	TF	WSCNNLSTM			Cell Line	TF	WSCNNLSTM		
		Max	Average	Noisy-and			Max	Average	Noisy-and
Gm12878	Batf	0.810	0.817	**0.833**	Gm12878	Pax5n19	0.748	0.753	**0.794**
Gm12878	Bcl1	0.661	0.665	**0.727**	Gm12878	Pbx3	0.790	0.801	**0.818**
Gm12878	Bcl3	0.797	0.795	**0.821**	Gm12878	Pou2	0.758	0.769	**0.781**
Gm12878	Bclaf	0.769	0.761	**0.799**	Gm12878	Pu1	0.926	0.927	**0.942**
Gm12878	Ebf	0.825	0.834	**0.850**	Gm12878	Rad21	0.975	0.965	**0.982**
Gm12878	Egr1	0.909	0.916	**0.940**	Gm12878	Sp1	0.668	0.681	**0.714**
Gm12878	Elf1	0.888	0.886	**0.897**	Gm12878	Srf	0.792	0.795	**0.850**
Gm12878	Ets1	0.826	0.859	**0.887**	Gm12878	Taf1	0.864	0.870	**0.878**
Gm12878	Irf4	0.673	0.656	**0.740**	Gm12878	Tcf12	0.855	0.857	**0.874**
Gm12878	Mef2a	0.607	0.607	**0.746**	Gm12878	Usf1	0.912	0.907	**0.929**
Gm12878	Nrsf	0.682	0.842	**0.877**	Gm12878	Yy1	0.893	0.899	**0.907**
Gm12878	Pax5c20	0.862	0.863	**0.879**	**Average PR AUC**		0.804	0.814	**0.846**
		Noisy-and vs Max					Noisy-and vs Average		
**P-value**		p-value $\leqslant$ 1.362e-04					p-value $\leqslant$ 3.553e-05		

Supplementary Table 7.3: F1-score of WSCNNLSTM on the 23 ChIP-seq datasets in Gm12878 cell line, and the best scores are highlighted in bold.

Cell Line	TF	WSCNNLSTM			Cell Line	TF	WSCNNLSTM		
		Max	Average	Noisy-and			Max	Average	Noisy-and
Gm12878	Batf	0.720	0.733	**0.747**	Gm12878	Pax5n19	0.653	0.652	**0.680**
Gm12878	Bcl1	0.545	0.561	**0.620**	Gm12878	Pbx3	0.697	0.704	**0.725**
Gm12878	Bcl3	0.696	0.690	**0.728**	Gm12878	Pou2	0.652	0.665	**0.661**
Gm12878	Bclaf	0.655	0.660	**0.685**	Gm12878	Pu1	0.870	0.874	**0.890**
Gm12878	Ebf	0.719	0.739	**0.754**	Gm12878	Rad21	0.930	0.916	**0.939**
Gm12878	Egr1	0.836	0.858	**0.882**	Gm12878	Sp1	0.542	0.569	**0.568**
Gm12878	Elf1	0.802	0.795	**0.812**	Gm12878	Srf	0.693	0.701	**0.749**
Gm12878	Ets1	0.751	0.783	**0.786**	Gm12878	Taf1	0.799	0.801	**0.806**
Gm12878	Irf4	0.535	0.538	**0.642**	Gm12878	Tcf12	0.766	0.769	**0.785**
Gm12878	Mef2a	0.483	0.489	**0.648**	Gm12878	Usf1	0.836	0.838	**0.854**
Gm12878	Nrsf	0.527	0.750	**0.800**	Gm12878	Yy1	0.800	0.805	**0.810**
Gm12878	Pax5c20	0.769	0.780	**0.782**	**Average F1-score**		0.708	0.725	**0.754**
		Noisy-and vs Max					Noisy-and vs Average		
**P-value**		p-value ⩽ 1.361e-03					p-value ⩽ 8.627e-04		
